# Time to surgical stabilization of rib fractures: does it impact outcomes?

**DOI:** 10.1136/tsaco-2023-001233

**Published:** 2024-07-11

**Authors:** Joseph D Forrester, Babak Sarani, Maximilian Peter Forssten, Yang Cao, Frank Hildebrand, Ahmad Mohammad Ismail, Marcelo A F Ribeiro Jr., Shahin Mohseni

**Affiliations:** 1 Stanford University, Stanford, California, USA; 2 Center of Trauma and Critical Care, The George Washington University, Washington, District of Columbia, USA; 3 School of Medical Sciences, Örebro University, Orebro, Sweden; 4 Department of Orthopedic Surgery, Örebro University Hospital, Orebro, Sweden; 5 Clinical Epidemiology and Biostatistics, School of Medical Sciences, Faculty of Medicine and Health, Orebro University, Orebro, Sweden; 6 Department of Orthopedics, Trauma, and Reconstructive Surgery, University Hospital RWTH Aachen, Aachen, Germany; 7 Department of Surgery, Sheikh Shakhbout Medical City, Abu Dabi, Abu Dhabi, UAE; 8 Division of Trauma, Critical Care & Acute Care Surgery, Department of Surgery, Sheikh Shakhbout Medical City, Abu Dabi, UAE

**Keywords:** rib fractures, Thoracic Surgical Procedures, Outcome Assessment, Health Care, Time-To-Treatment

## Abstract

**Objectives:**

Rib fractures are common, morbid, and potentially lethal. Intuitively, if interventions to mitigate downstream effects of rib fractures can be implemented early, likelihood of developing these complications should be reduced. Surgical stabilization of rib fractures (SSRF) is one therapeutic intervention shown to be useful for mitigating complications of these common fractures. Our aim was to investigate for association between time to SSRF and complications among patients with isolated rib fractures undergoing SSRF.

**Methods:**

The 2016–2019 American College of Surgeons Trauma Quality Improvement Program (TQIP) database was queried to identify patient >18 years with isolated thoracic injury undergoing SSRF. Patients were divided into three groups: SSRF ≤2 days, SSRF >2 days but <3 days, and SSRF >3 days. Poisson regression, and adjusting for demographic and clinical covariates, was used to evaluate the association between time to SSRF and the primary endpoint, in-hospital complications. Quantile regression was used to evaluate the effects of time to SSRF on the secondary endpoints, hospital and intensive care unit (ICU) length of stay (LOS).

**Results:**

Out of 2185 patients, 918 (42%) underwent SSRF <2 days, 432 (20%) underwent SSRF >2 days but <3 days, and 835 (38%) underwent SSRF >3 days. Hemothorax was more common among patients undergoing SSRF >3 days, otherwise all demographic and clinical variables were similar between groups. After adjusting for potential confounding, SSRF >3 days was associated with a threefold risk of composite in-hospital complications (adjusted incidence rate ratio: 3.15, 95% CI 1.76 to 5.62; p<0.001), a 4-day increase in total hospital LOS (change in median LOS: 4.09; 95% CI 3.69 to 4.49, p<0.001), and a nearly 2-day increase in median ICU LOS (change in median LOS: 1.70; 95% CI 1.32 to 2.08, p<0.001), compared with SSRF ≤2 days.

**Conclusion:**

Among patients undergoing SSRF in TQIP, earlier SSRF is associated with less in-hospital complications and shorter hospital stays. Standardization of time to SSRF as a trauma quality metric should be considered.

**Level of evidence:**

Level II, retrospective.

**WHAT IS ALREADY KNOWN ON THIS TOPIC**

- Time to surgical stabilization of rib fractures (SSRF) has been postulated to be associated with outcomes after surgery.

**WHAT THIS STUDY ADDS**

- Using 4 years of nationally representative data, shorter time to SSRFs was associated with improved outcomes after surgery.

**HOW THIS STUDY MIGHT AFFECT RESEARCH, PRACTICE OR POLICY**

- Time to SSRFs should be considered as a quality metric by bodies responsible for governing the provision of trauma care.

## Introduction

 Rib fractures are common, morbid, and potentially lethal injuries.^1^ For patients with flail chest the impacts of concomitant pulmonary contusion and gross chest wall instability can lead to prolonged intubation, pneumonia, need for tracheostomy, and death.^2 3^ Even among patients with a less severe fracture pattern, sequelae of pain from the fractures often compound over time as splinting leads to atelectasis and impaired cough, leading to hypoxemia or ventilation/perfusion mismatch, tachypnea, increased work of breathing, and ultimately pneumonia or respiratory failure.

Intuitively, if interventions to mitigate these downstream effects of rib fractures can be implemented early, the likelihood of developing these complications should be reduced. Surgical stabilization of rib fractures (SSRF) is one therapeutic intervention shown to be useful for mitigating complications of these common fractures in select patients.^1^ Yet ubiquitous utilization of early SSRF to reduce the morbidity and mortality of these fractures, once other immediately life-threatening pathological findings have been dealt with, remains elusive. The aim of our study was to investigate the association between time to SSRF and complications among patients with isolated rib fractures. Our hypothesis is earlier SSRF would be associated with decreased in-hospital complications, hospital and intensive care unit (ICU) length of stay (LOS).

## Methods

Data for the study were obtained from the 2016–2019 American College of Surgeons Trauma Quality Improvement Program (TQIP) database that included information on demographics, injury characteristics, surgical interventions, discharge outcomes, and complications.^4 5^ The study included only adult rib fracture patients (≥18 years) who suffered an isolated thoracic injury and underwent SSRF. The use of SSRF was identified using International Classification of Diseases, 10th Rev (ICD-10) procedure codes registered in TQIP. This included ICD-10 codes starting with 0PH1, 0PH2, 0PS104, 0PS134, 0PS144, 0PS204, 0PS234, and 0PS244. An isolated thoracic injury was defined as a thorax Abbreviated Injury Scale (AIS) Score ≥1 and an AIS ≤1 in all other regions. Patients were excluded if they had a thorax AIS of 6, which is typically considered non-survivable. The current study used an anonymized, retrospective, national data set for all analyses. The study was deemed to be exempt from institutional review board approval by the sponsoring institution. The investigation was conducted in accordance with the Declaration of Helsinki and Strengthening the Reporting of Observational Studies in Epidemiology (STROBE) guidelines.^6 7^

### Statistical analysis

In this study, patients were divided into three groups based on the time from admission to SSRF: surgery within 2 days (≤48 hours), surgery within 2–3 days (>48 and ≤72 hours), and surgery after 3 days (>72 hours). Continuous variables were summarized as medians and IQRs, and categorical variables were presented as counts and percentages. The Kruskal-Wallis test was used to assess statistical significance of differences between continuous variables, and χ^2^ test or Fisher’s exact test were applied to assess categorical variables, as appropriate. The primary outcome of interest was incidence of in-hospital complications, and the secondary outcome was hospital and ICU LOS.

A Poisson regression model was used to evaluate the association between time to SSRF and in-hospital complications, adjusting for available demographic and clinical covariates to minimize potential confounding. In this model, time to SSRF, age, sex, race, highest AIS Score in each region, flail chest, sternal fracture, pneumothorax, hemothorax, pulmonary contusion, year of admission, trauma center level, hypertension, history of myocardial infarction, congestive heart failure, history of peripheral vascular disease, cerebrovascular disease, dementia, chronic obstructive pulmonary disease, smoking status, chronic renal failure, diabetes mellitus, cirrhosis, bleeding disorder, currently receiving chemotherapy for cancer, disseminated cancer, drug use disorder, alcohol use disorder, major psychiatric illness, and advanced directives limiting care were included as explanatory variables, and in-hospital complications were set as the response variable. The complications of interest were myocardial infarction, cardiac arrest with cardiopulmonary resuscitation, stroke, deep vein thrombosis (DVT), pulmonary embolism, acute respiratory distress syndrome (ARDS), pneumonia, and surgical site infection. Results are presented as adjusted incidence rate ratios (IRRs) and corresponding 95% CIs. Overdispersion was checked based on the dispersion ratio calculated using the performance package. Robust standard errors, which are not overly influenced by the assumptions regarding residuals, were used to calculate the 95% CIs.^8^

A quantile regression model was used to evaluate the effects of time to SSRF on hospital as well as ICU LOS. The same covariates were included as in the Poisson regression model and results were presented as change in median ICU LOS along with corresponding 95% CIs.

A two-sided value of p<05 was considered statistically significant in all analyses. Multiple imputation by chained equations was used to handle missing data. All analyses were performed in the software R V.4.0.5 using packages tidyverse, haven, mice, robustbase, quantreg, and performance.

## Results

Out of 2185 patients meeting inclusion criteria, 918 (42%) underwent SSRF within 2 days, 432 (20%) underwent SSRF within 2–3 days, and 835 (38%) underwent SSRF after 3 days. There were no differences in age, race, comorbidities, or advanced directives limiting care between groups. Most patients were male and identified as white. The most common comorbidity was hypertension, followed by diabetes, and chronic obstructive pulmonary disease (table 1). There was no difference in the percentage of patients in each of the three treatment groups by year. Injury severity was similar when comparing patients’ thorax AIS as well as the presence of specific intrathoracic injuries (table 2). Although the percentage of patients with flail chest and pneumothorax were similar between groups (30%–35% and 66%–68%, respectively), hemothorax was slightly more prevalent among patients who underwent SSRF after 3 days, compared with those who underwent SSRF within 2 days or 2–3 days (crude incidence rates: 58.2% vs. 52.4% and 51.2%, p=0.017). The majority of patients had a thorax AIS Score ≥3. Sternal fractures were the least common concomitant injury across all groups (table 2). Intubation prior to SSRF was unable to be included as a covariate given that over 82% of patients were missing these data.

**Table 1:** Demographics of patients with surgically managed rib fractures

	Surgery within 2 days (n=918)	Surgery within 2–3 days (n=432)	Surgery after 3 days (n=835)	P value
Age, median (IQR), years	61 (51–71)	61 (53–72)	62 (51–72)	0.700
Sex, n (%)				0.643
Female	261 (28.4)	132 (30.6)	235 (28.1)	
Male	657 (71.6)	300 (69.4)	600 (71.9)	
Race, n (%)				0.169
White	792 (86.3)	368 (85.2)	712 (85.3)	
Black	55 (6.0)	28 (6.5)	69 (8.3)	
Asian	13 (1.4)	9 (2.1)	6 (0.7)	
American Indian	8 (0.9)	0 (0.0)	7 (0.8)	
Pacific islander	0 (0.0)	0 (0.0)	1 (0.1)	
Other	36 (3.9)	22 (5.1)	35 (4.2)	
Missing	3 (0.3)	0 (0.0)	3 (0.4)	
Hypertension, n (%)	411 (44.8)	196 (45.4)	410 (49.1)	0.166
Previous myocardial infarction, n (%)	7 (0.8)	1 (0.2)	10 (1.2)	0.203
Congestive heart failure, n (%)	24 (2.6)	14 (3.2)	33 (4.0)	0.288
History of peripheral vascular disease, n (%)	6 (0.7)	1 (0.2)	5 (0.6)	0.709
Cerebrovascular disease, n (%)	23 (2.5)	12 (2.8)	26 (3.1)	0.742
Dementia, n (%)	24 (2.6)	13 (3.0)	27 (3.2)	0.740
COPD, n (%)	92 (10.0)	55 (12.7)	110 (13.2)	0.097
Current smoker, n (%)	210 (22.9)	113 (26.2)	233 (27.9)	0.051
Chronic renal failure, n (%)	10 (1.1)	7 (1.6)	10 (1.2)	0.706
Diabetes mellitus, n (%)	141 (15.4)	64 (14.8)	156 (18.7)	0.098
Cirrhosis, n (%)	9 (1.0)	4 (0.9)	10 (1.2)	0.890
Coagulopathy, n (%)	25 (2.7)	7 (1.6)	17 (2.0)	0.388
Drug use disorder, n (%)	41 (4.5)	24 (5.6)	59 (7.1)	0.063
Alcohol use disorder, n (%)	70 (7.6)	32 (7.4)	86 (10.3)	0.084
Major psychiatric illness, n (%)	97 (10.6)	42 (9.7)	88 (10.5)	0.879
Advanced directive limiting care, n (%)	24 (2.6)	14 (3.2)	17 (2.0)	0.418

COPDchronic obstructive pulmonary disease

**Table 2:** Clinical characteristics of patients with surgically managed rib fractures

	Surgery within 2 days (n=918)	Surgery within 2–3 days (n=432)	Surgery after 3 days (n=835)	P value
Head AIS, n (%)				0.497
Injury not present	838 (91.3)	401 (92.8)	759 (90.9)	
1	80 (8.7)	31 (7.2)	76 (9.1)	
Face AIS, n (%)				0.648
Injury not present	825 (89.9)	382 (88.4)	741 (88.7)	
1	93 (10.1)	50 (11.6)	94 (11.3)	
Neck AIS, n (%)				0.596
Injury not present	911 (99.2)	431 (99.8)	830 (99.4)	
1	7 (0.8)	1 (0.2)	5 (0.6)	
Spine AIS, n (%)				0.831
Injury not present	912 (99.3)	429 (99.3)	831 (99.5)	
1	6 (0.7)	3 (0.7)	4 (0.5)	
Thorax AIS, n (%)				0.389
2	34 (3.7)	19 (4.4)	33 (4.0)	
3	677 (73.7)	333 (77.1)	606 (72.6)	
4	189 (20.6)	72 (16.7)	171 (20.5)	
5	18 (2.0)	8 (1.9)	25 (3.0)	
Abdomen AIS, n (%)				0.381
Injury not present	861 (93.8)	402 (93.1)	769 (92.1)	
1	57 (6.2)	30 (6.9)	66 (7.9)	
Upper extremity AIS, n (%)				0.773
Injury not present	759 (82.7)	351 (81.2)	691 (82.8)	
1	159 (17.3)	81 (18.8)	144 (17.2)	
Lower extremity AIS, n (%)				0.862
Injury not present	782 (85.2)	370 (85.6)	706 (84.6)	
1	136 (14.8)	62 (14.4)	129 (15.4)	
External/other AIS, n (%)				0.517
Injury not present	857 (93.4)	410 (94.9)	786 (94.1)	
1	61 (6.6)	22 (5.1)	49 (5.9)	
Flail chest, n (%)	325 (35.4)	131 (30.3)	287 (34.4)	0.177
Sternal fracture, n (%)	33 (3.6)	18 (4.2)	47 (5.6)	0.114
Pneumothorax, n (%)	609 (66.3)	288 (66.7)	569 (68.1)	0.709
Hemothorax, n (%)	481 (52.4)	221 (51.2)	486 (58.2)	0.017
Pulmonary contusion, n (%)	286 (31.2)	145 (33.6)	286 (34.3)	0.361
Trauma center level, n (%)				<0.001
I	412 (44.9)	175 (40.5)	337 (40.4)	
II	341 (37.1)	162 (37.5)	250 (29.9)	
III	21 (2.3)	9 (2.1)	32 (3.8)	
Not verified/designated	144 (15.7)	86 (19.9)	216 (25.9)	

AISAbbreviated injury Scale

Before adjusting for confounders, patients who underwent SSRF after 3 days exhibited higher overall rates of any complication compared with those who underwent SSRF within 2 days or 2–3 days (8% vs. 3% and 4%, p<0.001). When compared with those patients operated on within 2 days patients who underwent SSRF after 3 days had higher rates of myocardial infarction (0% vs. 1%, p=0.014), DVT (1% vs. 2%, p=0.008), ARDS (0% vs. 1%, p=0.006), and pneumonia (0% vs. 2%, p=0.003). Total hospital LOS also tended to be longer in patients who underwent SSRF after 3 days compared with those who underwent SSRF within 2 days or 2–3 days (medians: 12 days vs. 8 days and 9 days, p<0.001). Although rates of ICU admission among cohorts were similar, patients who underwent SSRF after 3 days tended to require a longer ICU stay compared with those who underwent SSRF within 2 days or 2–3 days (medians: 6 days vs. 4 days and 5 days, p<0.001) (table 3). After adjusting for potential confounding, SSRF more than 3 days after admission was associated with an almost threefold increased odds of composite in-hospital complications (adjusted IRR: 2.44; 95% CI 1.55 to 3.83, p<0.001), a roughly 4-day increase in total hospital LOS (change in median LOS: 3.99; 95% CI 3.55 to 4.43, p<0.001), and an approximately 2-day increase in the median ICU LOS (change in median LOS: 1.73; 95% CI 1.27 to 2.19, p<0.001), compared with SSRF within 2 days (table 4). Overdispersion was not present in the Poisson regression model (dispersion ratio: 0.852, p=1.00).

**Table 3:** Crude outcomes in patients with surgically managed rib fractures

	Surgery within 2 days (n=918)	Surgery within 2–3 days (n=432)	Surgery after 3 days (n=835)	P value
Time to surgery, median (IQR)	1.2 (0.76–1.7)	2.6 (2.3–2.8)	4.7 (3.7–6.2)	<0.001
Any complication, n (%)	26 (2.8)	16 (3.7)	67 (8.0)	<0.001
Myocardial infarction, n (%)	0 (0.0)	0 (0.0)	5 (0.6)	0.014
Cardiac arrest with CPR, n (%)	8 (0.9)	2 (0.5)	9 (1.1)	0.606
Stroke, n (%)	2 (0.2)	0 (0.0)	0 (0.0)	0.690
DVT, n (%)	5 (0.5)	6 (1.4)	19 (2.3)	0.008
Pulmonary embolism, n (%)	8 (0.9)	1 (0.2)	6 (0.7)	0.495
ARDS, n (%)	2 (0.2)	3 (0.7)	12 (1.4)	0.006
Pneumonia, n (%)	4 (0.4)	2 (0.5)	16 (1.9)	0.003
Surgical site infection, n (%)	2 (0.2)	2 (0.5)	9 (1.1)	0.053
In-hospital mortality, n (%)	12 (1.3)	9 (2.1)	11 (1.3)	0.489
Hospital length of stay, median (IQR)	8.0 (6.0–10)	9.0 (7.0–11)	12 (9.0–16)	<0.001
Missing, n (%)	6 (0.7)	5 (1.2)	7 (0.8)	
ICU admission, n (%)	557 (60.7)	267 (61.8)	544 (65.1)	0.143
ICU length of stay, median (IQR)	4.0 (3.0–6.0)	5.0 (3.0–8.0)	6.0 (3.0–10)	<0.001

Length of stay is measured in days.

ARDSacute respiratory distress syndromeCPRcardiopulmonary resuscitationDVTdeep vein thrombosisICUintensive care unit

**Table 4:** Association between time to surgery and morbidity and ICU length of stay in patients with surgically managed rib fractures

Outcome	Surgery within 2 days	Surgery within 2–3 days	Surgery after 3 days
**IRR (95% CI**)	**P value**	**IRR (95% CI**)	**P value**
In-hospital complications	Reference	1.21 (0.65 to 2.24)	0.554	2.44 (1.55 to 3.83)	<0.001
**Change in median (95% CI**)	**P value**	**Change in median (95% CI**)	**P value**
Hospital length of stay	Reference	1.00 (0.57 to 1.43)	<0.001	3.99 (3.55 to 4.43)	<0.001
ICU length of stay	Reference	1.04 (0.43 to 1.65)	<0.001	1.73 (1.27 to 2.19)	<0.001

IRRs are calculated using a Poisson regression model with robust SEs. Change in median length of stay is calculated using a quantile regression model. Length of stay is measured in days. Missing values were managed using multiple imputation by chained equations. All analyses were adjusted for age, sex, race, highest Abbreviated Injury Severity Score in each region, flail chest, sternal fracture, pneumothorax, hemothorax, pulmonary contusion, year of admission, trauma center level, hypertension, history of myocardial infarction, congestive heart failure, history of peripheral vascular disease, cerebrovascular disease, dementia, chronic obstructive pulmonary disease, smoking status, chronic renal failure, diabetes mellitus, cirrhosis, bleeding disorder, currently receiving chemotherapy for cancer, disseminated cancer, drug use disorder, alcohol use disorder, major psychiatric illness, and advanced directives limiting care.

ICUIntensive care unitIRRincidence rate ratio

## Discussion

Existing studies evaluating time to surgery for SSRF have demonstrated that odds of complications after SSRF tend to be lower for patients on whom SSRF is performed earlier.^9^,^13^ Although variability exists in what is considered ‘early’, ranging from <24 hours^9^ to <6 days,^11^ the trend is clear. Patients on whom SSRF is performed sooner appear to have lower odds of pneumonia,^9^ shorter duration of mechanical ventilation,^9 11 12^ lower risk of tracheostomy,^9^ and, not surprisingly, generally have shorter hospital stays, ICU stays^10 12^ and lower cost.^11 12^ Our study—the largest evaluation to date evaluating the influence of time to SSRF on in-hospital adverse outcomes and LOS—generally corroborates these prior findings.^9^,^13^ When SSRF is performed later after admission, the odds of an in-hospital complication increase as does the length of hospitalization.

Yet an elusive question remains unanswered; why do patients who undergo SSRF early appear to have better outcomes? It is possible that selection bias is driving the observed outcomes. Healthier patients with lower concomitant injury burden may be deemed by surgeons to tolerate an operation ‘better’ so are more likely to be in the early SSRF group. Conversely, some centers or surgeons may wait until a patient develops downstream sequelae of untreated or undertreated rib fractures prior to considering them for SSRF—increasing the chances of those patients developing complications, as much from the underlying rib fractures as the delayed surgery itself. However, in our analyses the groups appear equivalent with respect to their underlying comorbidities and are homogenous with respect to their injury burden. Other factors that could increase time to an operation include operating room or surgeon availability, surgeon expertise, equipment and equipment company representative availability, among other logistical issues. Finally, one additional potential explanation is also possible—that by providing an intervention early, an intervention capable of mitigating downstream effects of the pathological findings associated with rib fractures,^1^ patients do better.^13^ The concept of early fracture stabilization among patients with spine, acetabular, pelvic, femur, and tibia fractures is well established.^13^ So much so that institutional treatment guidelines detailing expected time to the operating room for these fractures is required for trauma center verification.^14^ However, this is not yet the case for flail chest or severe chest wall injury.

As suggested by some authors there may be a time threshold after which the residual benefit of SSRF becomes outweighed by potential complications.^15^ Unfortunately, as we did not include a cohort of patients who did not undergo SSRF, we cannot comment directly on this threshold, if present. Yet encouragingly, even though odds of in-hospital complications appear to increase as the time to SSRF increases, the overall rates of these complications in patients undergoing SSRF remain low. With the risk of any given complication after SSRF in the single digits even among patients who underwent SSRF after 3 days, it appears that the surgery remains relatively well tolerated overall, a finding consistent with prior studies.^16^,^19^

There are several important limitations to this study. First, the data in TQIP are retrospective and there exists opportunity for selection bias. This is compounded by heterogenous practice patterns among application of SSRF for injured patients with rib fractures. Additionally, during the evaluated time frame, the indications for SSRF expanded to include non-flail rib fractures in the presence of certain physiologic indicators of impaired ventilatory mechanics. This variability in practice pattern over time, as well as provider and center heterogeneity were unable to be accounted for in our analysis as these descriptors are not available within TQIP. This may be particularly pronounced given variation in application of selection criteria to determine candidates for SSRF. Second, there could be misclassification bias as coders may interpret in-hospital outcomes differently at the institutional or regional level. Third, we were unable to include preoperative intubation as a covariate due to degree of missingness. Need for preoperative intubation could be a useful marker of underlying injury which could confound our analysis. Fourth, there is considerable variability among what is considered early SSRF. This lack of a standard definition limits comparisons between studies. Fifth, SSRF techniques have changed considerably in the last decade. As less invasive methods of rib stabilization have become available it is possible the observed increase in complications with delayed SSRF will become less pronounced. Year of operation was not included in analyses, limiting interpretation of this potential effect. Sixth, generalizability of these findings to populations outside of the USA should be performed with caution given the source of the TQIP data.

## Conclusions

Among patients undergoing SSRF in TQIP, earlier rib stabilization is associated with lower in-hospital complications and shorter hospital stays. Overall rates of in-hospital complications after SSRF remain low. Standardization of time to SSRF as a trauma quality metric should be considered.

## Data Availability

Data are available upon reasonable request.
